# Age is reflected in the Fractal Dimensionality of MRI Diffusion Based Tractography

**DOI:** 10.1038/s41598-018-23769-6

**Published:** 2018-04-03

**Authors:** Gernot Reishofer, Fritz Studencnik, Karl Koschutnig, Hannes Deutschmann, Helmut Ahammer, Guilherme Wood

**Affiliations:** 1 Medical University of Graz, Department of Radiology, Division of Neuroradiology, Vascular and Interventional Radiology, Graz, Austria; 2 University of Graz, Department of Psychology, Graz, Austria; 3 Medical University of Graz, Institute of Biophysics, Graz, Austria

## Abstract

Fractal analysis is a widely used tool to analyze the geometrical complexity of biological structures. The geometry of natural objects such as plants, clouds, cellular structures, blood vessel, and many others cannot be described sufficiently with Euclidian geometric properties, but can be represented by a parameter called the fractal dimension. Here we show that a specific estimate of fractal dimension, the correlation dimension, is able to describe changes in the structural complexity of the human brain, based on data from magnetic resonance diffusion imaging. White matter nerve fiber bundles, represented by tractograms, were analyzed with regards to geometrical complexity, using fractal geometry. The well-known age-related change of white matter tissue was used to verify changes by means of fractal dimension. Structural changes in the brain were successfully be observed and quantified by fractal dimension and compared with changes in fractional anisotropy.

## Introduction

In the late sixties Benoit Mandelbrot developed a mathematical concept to describe geometrical structures, denoted as fractals, whose measured metric properties (length, area or volume) depend on the scale of measurement. A fractal can be defined as a set, whose fractal dimension (*FD*) is a non-integer value between the topological dimension, which is zero for a point, one for a curve and two for a plane, and its embedding dimension. With this, *FD* can describe geometrical features such as self-similarity or space-filling properties of textures or structures that are obtained by stochastic processes. In his pioneering work “How long is the coast of Britain^1?”^ Mandelbrot paved the way for the field of fractal analysis, a mathematical framework describing geometrical structures that cannot be characterized sufficiently with Euclidian geometry. For a natural structure or an image, the fractal dimension cannot be calculated exactly, but is usually approximated as the ratio of change in detail with change in scale, plotted in a double logarithmic plot where the slope provides an estimate of *FD*.

In the last two decades, a huge variety of applications utilizing fractal analysis have been published on different biomedical fields from the analysis of DNA base sequences^2^ and the classification of biological structures^3^ to the description of microvascularity in gliomas^4,5^. The complex structures of the brain have been extensively investigated using fractal methods focusing on the brain’s surface^6^, the geometrical complexity of white matter^7^, changes in the structure of the cerebrovascular system^8^ due to pathologies and for analyzing the complex structure of neural networks^9,10^. Fractal analysis of the brain’s geometrical structure was correlated with pathologies such as Multiple Scleroses^11,12^ or Alzheimer’s disease^13^, suggesting that structural alterations, specifically in white matter structure, can be captured by changes in *FD*. Considering these studies, *FD* seems to be a sensitive biomarker for changes in the geometrical complexity of the brain.

Structural changes of the brain, that concern all of us, are due to a normal aging process and effects the brain on many levels including vascularization^14^, atrophy^15^, changes in cortical and subcortical regions^16,17^ and changes in myelination. Cerebral white matter is subject to structural changes during the entire life-span, which has been shown in several MRI studies^18,19^. Diffusion tensor imaging revealed that the fractional anisotropy (FA), a measure for the anisotropy of the diffusion process, increases in the first three decades and decreases in the following decades^19–22^. However, age-related changes of the brain structure are closely linked to mental fitness and cognitive performance^23^. Hence, a quantification of structural changes is of highest interest.

**Figure 1:**
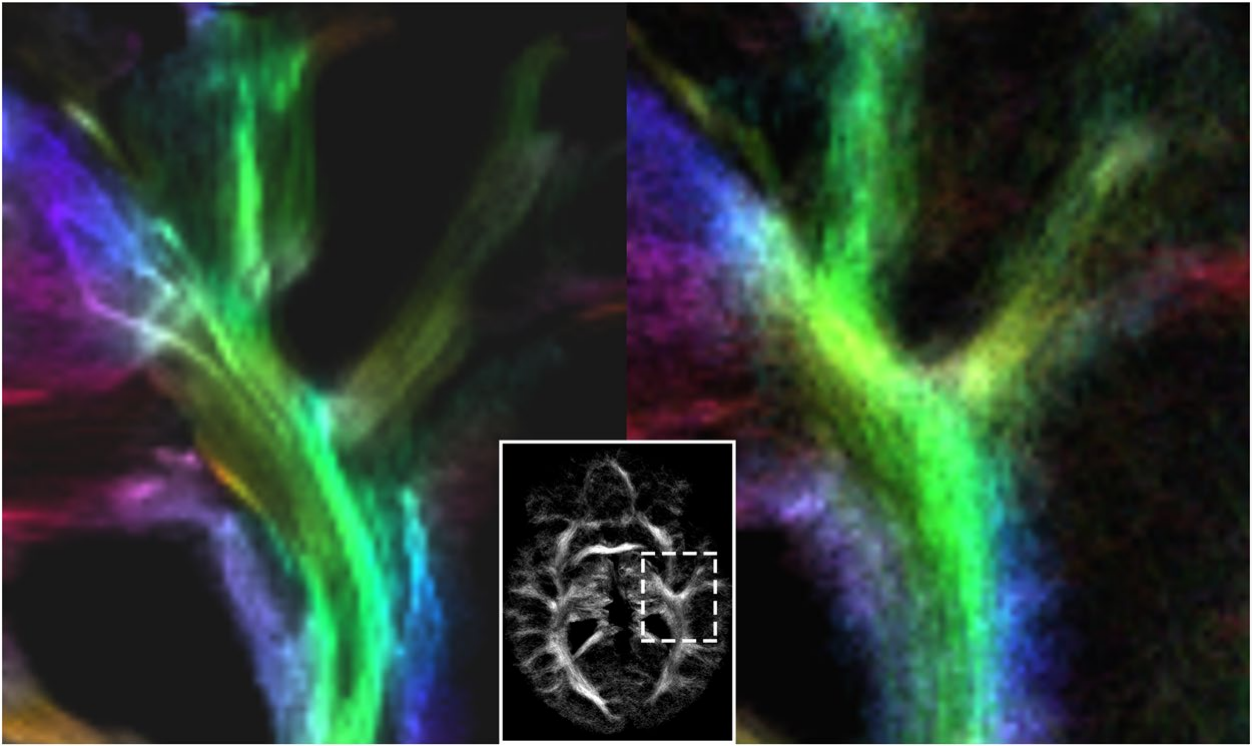
Comparison of fiber tracts based on DTI (left) and based on CSD (right). The images demonstrate the more realistic presentation of white matter tracts when crossing fiber configurations are taken into account. Tensor based tractography my lead to an unnatural fiber density (marked with white arrows). The more natural representation of cerebral white matter, obtained with CSD, served as input for fractal analysis.

When analyzing changes in FD of white matter structure, two strategies are mainly used. Firstly, fractal analysis of the entire binary WM mask^24^, which is directly obtained from a T_1_-weighted structural Magnetic Resonance Imaging (MRI) scan. Secondly, the fractal dimension is obtained from a skeletonized version of a T_1_-weighted white matter mask^25^. However, due to limitations in spatial resolution, caused by MRI basic conditions such as signal strength and scan time, both methods are only rough approximations to investigate the underlying complex structure of nerve fiber bundles. The most accurate macroscopic geometrical representation of the neural structure of the brain, by means of MRI, is provided by diffusion tensor imaging (DTI) based fiber tracking^26^ and its further developments. These methods allow for the visualization of neural fiber tracts based on a direction-sensitive MR measurement of the diffusion of free water. The sum of all fiber tracts represented by lines in a three dimensional space and color-coded according to their orientation is usually referred to as tractogram. Fractal properties of MR tractograms have been firstly investigated by Katsaloulis *et al*.^27,28^. However, studies including large cohorts of subjects or patients with neurological pathologies are missing. One reason might be that the diffusion tensor model, which is widely used for tractography, is not able to resolve complex fiber configurations such as crossing or kissing fibers. Then, fractal analysis describes space-filling properties without capturing the full geometrical complexity and group differences may remain unrevealed. New developments in diffusion data analysis account for complex fiber orientations and provide tractograms with higher accuracy, more suitable for fractal analysis (Fig. 1). Methods like q-ball imaging^29^, diffusion spectrum imaging^30^, or constrained spherical deconvolution (CSD)^31^ have been developed for this purpose but require a large number of diffusion sensitizing gradient directions (60 and more) making the MR measurement challenging with respect to scan time and signal-to-noise ratio. However, analyzing tractograms inherit a profound advantage over analyzing image data. While images are limited in resolution due to the sampled imaging matrix, tractograms are defined by points in space, referred to as fulcrums, that allows for a discretization with arbitrary resolution. Given that, for reliable estimates of FD, a structure to be analyzed with fractal analysis should obey scaling rules over several scales^32^, tractograms might be ideal candidates for fractal analysis of cerebral white matter.

There are several ways to calculate *FD* for a natural structure or for an image. All of these are approximations of the Hausdorff Dimension^33^, a single value, characterizing the geometrical complexity of a structure. The most prominent method that is often used due to its simplicity and computational efficiency is called the Box-counting method^34^ providing a value of *FD* called the Box-counting dimension^35^. However, fractal systems in nature are often insufficiently described by such a single value because self-similarity only exists in a limited number of magnitudes. Hence, a continuous spectrum of fractal dimensions instead of a single fractal dimension may provide a better geometrical description of natural complex structures such as tractograms. This characteristic, usually denoted as multifractality^36^, is considered when evaluating generalized fractal dimensions or Renyi dimensions.

In this proof of concept, we investigated, if *FD* captures age-related structural changes in white matter by analyzing tractograms obtained from CSD. Changes of *FD* values, obtained by multifractal analysis (MFA), are compared to changes of FA values and their age dependence is discussed. We demonstrate that fractal dimension is able to capture age related changes in white matter structure by analyzing 85 healthy subjects between eighteen and eighty-one years. We hypothesized that age dependent changes are more accurately modeled using the *FD* approach analyzing tractograms obtained from CSD compared to FA analysis based on the diffusion tensor model.

## Results

### Evaluation of the multifractal spectrum

**Figure 2:**
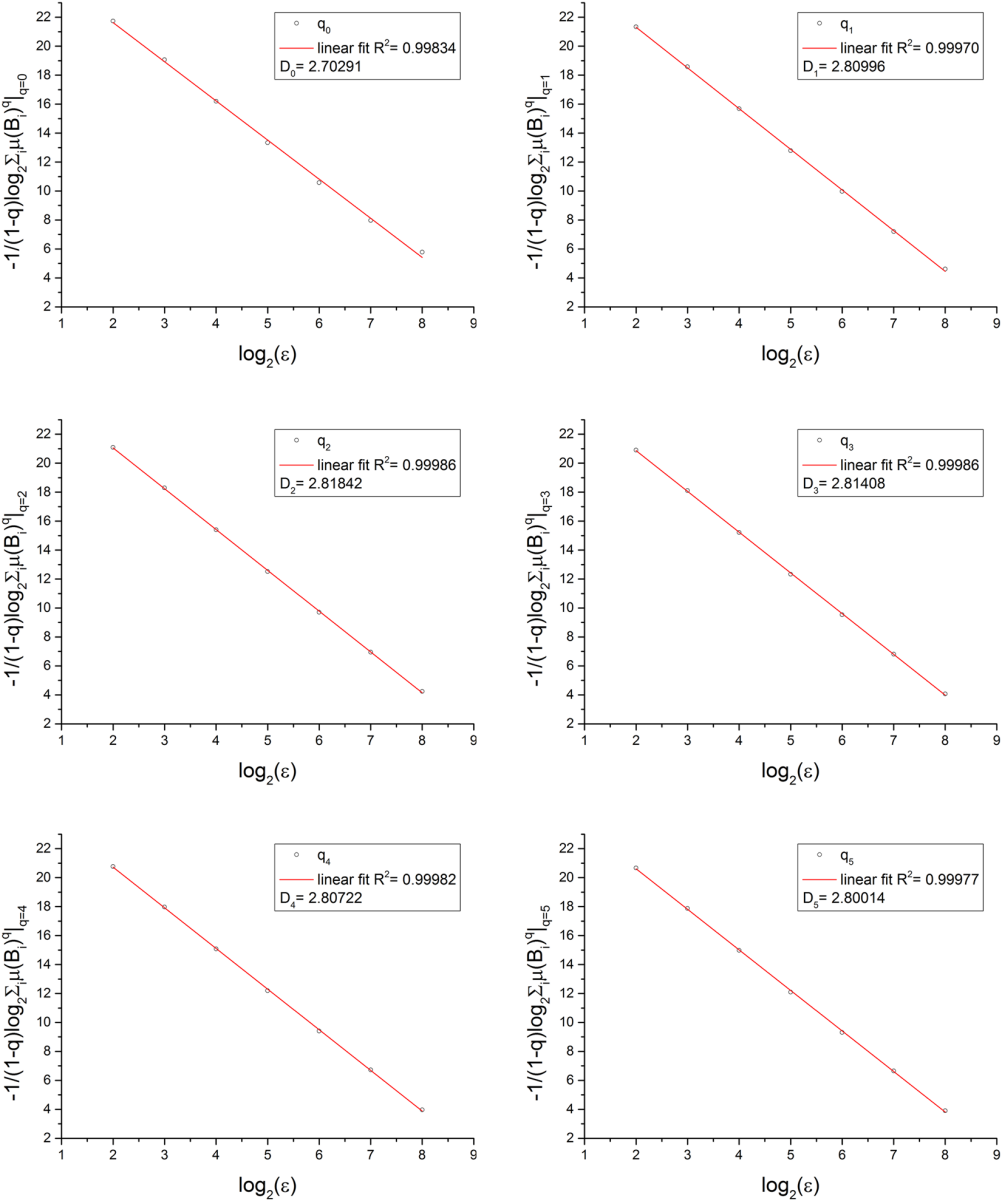
The absolute value of the slope in the double-logarithmic plot provides the fractal dimensions for the generalized dimensions or Renyi dimensions in the range q = [0 … 5]. The highest R^2^ was found for q = 2, indicating that the correlation dimension D_2_ is the best fractal estimator (highlighted by the green frame). Data are shown for one arbitrary chosen subject.

**Figure 3:**
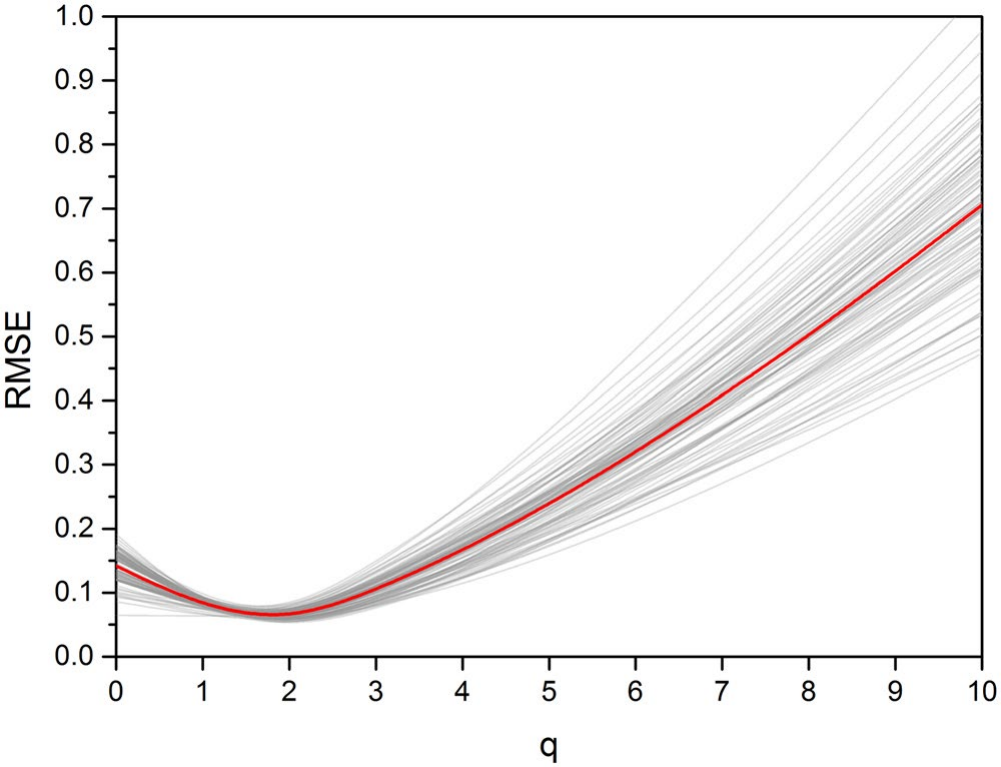
RMSE of the linear fit in the double-logarithmic plot. A minimum for RMSE was detected at q = 2 for all subjects. This demonstrates the stability of D_2_ as the superior *FD* estimator for all subjects. The red line indicates the mean value.

**Figure 4:**
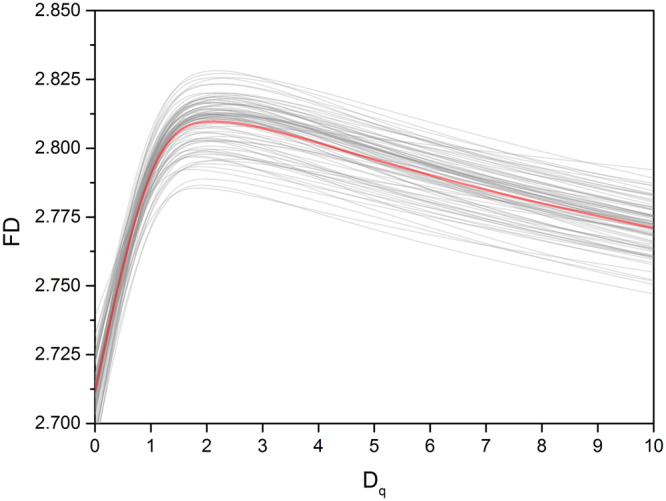
Generalized fractal dimensions in the range q = [0 … 10]. The fractal dimension shows a maximum for D_2_ with a monotonic decrease for D_q_ with q > 2. Please note that for ideal or geometric fractals D_q_ decreases for q ≥ 0. The red line indicates the mean value.

The generalized fractal dimensions D_q_ were evaluated as the slopes in the double-logarithmic plots $-1/(1-q)\mathrm{log}\,\sum _{i}\mu {({B}_{i})}^{q}$ against $\mathrm{log}(\varepsilon )$ for q = [0…10] (q = 0 … 5 shown in Fig. 2). The quality of the fit was indicated by the root mean square error (RMSE). RMSE felled from D_0_ (mean RMSE: 0.14 ± 0.02) until D_2_ (mean RMSE: 0.054 ± 0.006) and rose with increasing q, given the highest RMSE at D_10_ (mean RMSE: 0.7 ± 0.1) (Fig. 3). For all subjects, D_2_ showed the best linear relation, indicated by the minimum RMSE, and was therefore the preferred fractal estimator. Values of the generalized fractal dimensions D_q_ showed their highest values for D_2_ (mean D_2_: 2.811 ± 0.009). D_0_ and D_1_ were lower than D_2_ and for q > 2 the fractal dimension decreased monotonically. This is not in agreement with Eq. [4] because multifractal properties of the fractal spectrum are only given for generalized fractal dimensions D_q_ with q > 2. This observation was consistent for all subjects (Fig. 4).

### Age dependence of fractal dimension

**Figure 5:**
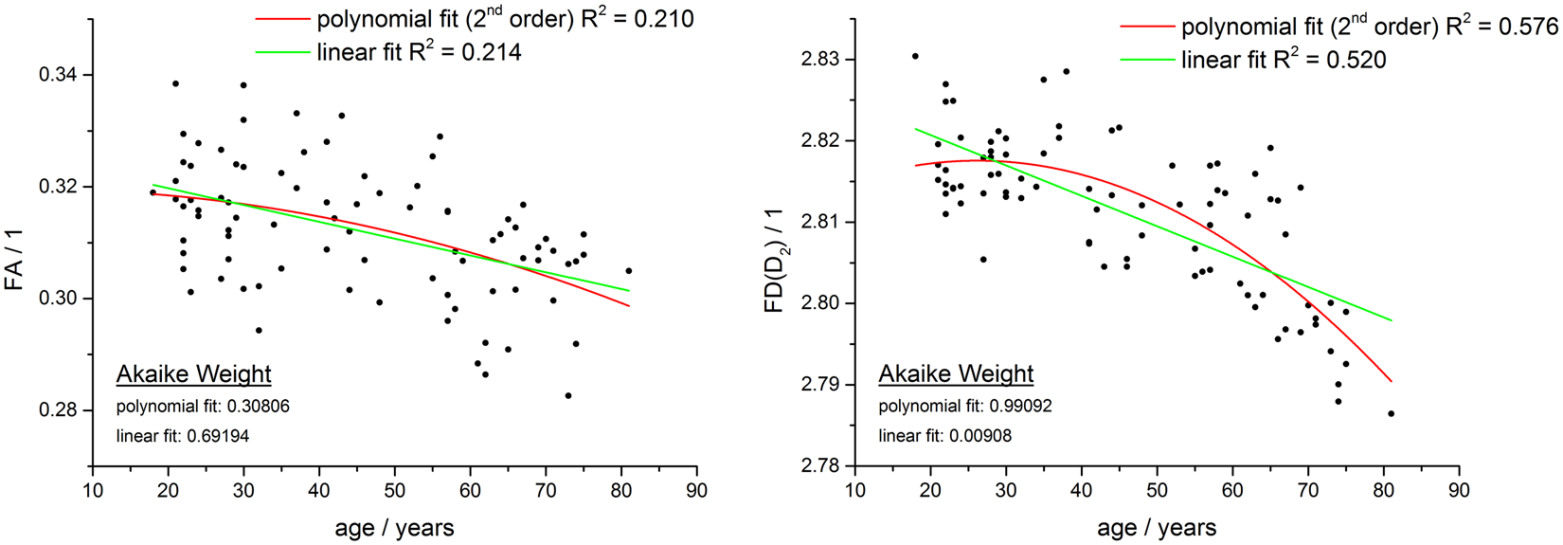
Correlation between FA and FD with age. Both parameters FA and *FD* show a negative correlation with subject’s age. While Akaike information criterion suggests a linear model (green line) for FA-age correlation, for *FD* the polynomial fit of 2^nd^ order (red line) is more probable. The negative correlation of D_2_ with age is better compared to FA-age correlation.

A correlation analysis between the generalized fractal dimensions (D_q_: q = [0…10] with age identified the highest correlation for D_2_, the correlation dimension (Table 1). The box-counting dimension did not correlate with age (p < 0.71). Both parameters, *FD* (D_2_) and FA, were reduced with increasing age (Fig. 5). Since the existence of non-linear age-related effects is well documented^21,37,38^, linear regression and additional polynomial regression was employed to describe the effect of age on FA and FD. In both cases, R^2^ was higher for *FD* than for FA (linear fit *FD*: R^2^ = 0.520, linear fit FA: R^2^ = 0.214; polynomial fit *FD*: R^2^ = 0.576, polynomial fit FA: R^2^ = 0.210). The Akaike Information Criterion (AIC) identified the polynomial fit as the preferred model for *FD* and the linear model for FA. The Akaike weights (AW) can be interpreted as the probability for a specific model fit and was evaluated as follows: *FD*: linear fit AW = 0.00908, polynomial fit AW = 0.99092; FA: linear fit AW = 0.69194, polynomial fit AW = 0.30806. This means that for *FD* the polynomial fit was about 109 times more likely to be correct than the linear fit and for FA the linear model was about 2 times more likely to be correct than the polynomial fit.

**Table 1:** Correlation between age and the generalized fractal dimensions (D_q_: q = [0…10]). Regression parameters and ANOVA results are presented for the polynomial fit of 2^nd^ order. n=85, p < 0.05. The correlation dimension D_2_ shows the best correlation with age.

D	Residual Sum of Squares	Adj. R-Square	ANOVA		
			Sum of Squares	F Value	Prob > F
**D**_**0**_	0.00823	−0.016	6.90595E-5	0.34	0.71
**D**_**1**_	0.00231	0.518	0.00261	46.19	3.66E-14
**D**_**2**_	0.00313	0.576	0.00442	57.98	2.22E-16
**D**_**3**_	0.00365	0.541	0.00451	50.59	4.88E-15
**D**_**4**_	0.00402	0.482	0.00393	40.11	7.12E-13
**D**_**5**_	0.00433	0.417	0.00328	31.08	8.96E-11
**D**_**6**_	0.00462	0.355	0.00272	24.12	5.78E-9
**D**_**7**_	0.00490	0.298	0.00226	18.87	1.81E-7
**D**_**8**_	0.00519	0.249	0.00189	14.93	2.95E-6
**D**_**9**_	0.00547	0.207	0.0016	11.98	2.73E-5
**D**_**10**_	0.00576	0.172	0.00137	9.76	1.573E-4

### Influence of sex, brain volume and mean fiber length

Both, *FD* (D_2_) and FA did not significantly differ between male and female subjects (D_2_: p < 0.84, FA: p < 0.19). To ensure that the age dependence of fractal dimension was not biased by brain volume changes due to normal aging processes, a regression analysis was performed. Given that female and male white matter volume significantly (p < 4.3·10^-8^) differed in volume (female: 501.9 ml ± 45.1 ml, male: 563.2 ml ± 47.2 ml), the regression analysis was performed separately for female and for male subjects. At the p = 0.05 level, the slopes were not significantly different from zero for both, female subjects (p < 0.98) and male subjects (p < 0.66) indicating that no correlation between white matter volume and age was observed in our data. Furthermore, we investigated if there was an age dependent change in mean fiber length that might influence the age dependence of the fractal dimensions. Female and male subjects were significantly different in mean fiber length at the p = 0.05 level (female: 66.8 mm ± 3.1 mm, male: 68.2 mm ± 2.9 mm) hence regression analysis was performed separately for female and male subjects. At the p = 0.05 level, the slopes were not significantly different from zero for female subjects (p < 0.1) and for male subjects (p < 0.65) supporting the view that no correlation between mean fiber length and age was observed in our data.

### Our main results can be summarized as follows

- The correlation dimension D_2_ is the preferred fractal dimension for analyzing tractograms.
- For the multifractal spectrum D_q_, D_2_ correlates best with age.
- Box-counting dimension D_0_ does not correlate with age.
- A polynomial model of 2^nd^ order describes better the correlation of D_2_ with age than a linear model.
- The correlation of D_2_ with age is better than the correlation of FA with age
- White matter brain volume and mean fiber length do not significantly correlate with age in our data.

## Discussion

For the first time, fractal analysis of tractograms described structural changes in human cerebral white matter due to healthy aging. The observed correlation of *FD*, specifically the correlation dimension D_2_, with age was much stronger compared to the correlation of FA with age. This can be explained by the fact that our *FD* calculation relied on much richer information than FA calculation. Firstly, FA is evaluated from the eigenvalues of the diffusion tensor which neglect the fiber orientation and only account for restricted diffusion strength. Secondly, FA is based on a tensor model that does not consider complex fiber configurations such as crossing or kissing fibers. Given that most regions of the brain contain such complex structures^39,40^, higher angular resolution tracking methods such as CSD^31,40^ provide a more reliable representation of the fiber nerves and are therefore more suitable to investigate structural changes. Other studies that used fractal analysis to investigate age-related white matter changes relied on the analysis of white matter shape^7,24,41^. Based on T_1_-weighted MR images with a typical matrix size of 256 × 256, the white matter was segmented and analyzed utilizing the box-counting dimension. We learned from these studies, that the white matter shape tends to reduce its complexity with increasing age, indicated by a decrease of *FD*. However, the shape of a white matter mask provides limited information about the entire white matter because the inner structure remains hidden. A much more comprehensive representation was given by the tractograms that were used in our study. Another benefit of analyzing tractograms is that image resolution is not a limiting factor for fractal analysis. It is well known that a fractal structure should obey scaling rules over several scales^32^. The assumption of this precondition is often violated due to small image matrixes. A tractogram consists of curves that are defined by fulcrums in a three dimensional space and with increasing number of curves the evaluated tractograms becomes denser. Such an object can be discretized with arbitrary high resolution and is independent of the matrix size of underlying DWI scan. With this, it was assured that image resolution was high enough for fractal analysis. In medical imaging, a similar technique is usually referred to as super-resolution track density imaging^42–44^.

Interestingly, the most frequently used measurement of *FD*, the box-counting dimension or capacity dimension D_0_ did not show any significant age dependence when applied on tractograms. The reason is that D_0_ is not sensitive to the density of points or more specifically to changes in density of points (see Eq. 3). The number of non-empty boxes is counted, regardless of the number of points within a box. If a structure is dense enough, even a small box may always contain a point and changes cannot be captured. Age dependence of *FD* was therefor only observable for D_q_, with q > 0 where the point density was taken into account. The best correlation of *FD* with age was found for D_2_. This is interesting in the light that for D_2_, estimated through the double logarithmic plot for q = 2, RMSE was smallest. So, for all subjects D_2_ gave the best estimate for *FD*. The highest value of *FD* was consistently found for D_2_ with absolute values close around 2.8 which is in line with previous obserevations^27^. This is not in line with observations for ideal or geometric fractals where the multifractal spectrum D_q_ versus q is a monotonic decreasing curve. However, in case of stochastic or finite fractals, anomalous multifractal spectra have been reported for aggregated particles^45^, for bit strings^46^, for boundaries of neuronal cells^47^ and for some mathematical fractals represented in digital images^32^. The origin of such curve shapes has not been investigated sufficiently and still is subject of scientific debate, but it is conceivable that scaling rules are only valid for specific ranges of the multifractal spectrum.

The method proposed in this proof-of-concept describes the geometric complexity of neural fiber tracts with a single parameter. The observed trend of decreasing *FD* with age was also found for FA and is congruent with other studies^16^. Although it is fascinating that a single value is definitely linked with structural changes in the brain, a closer look on specific regions might be in the scientific focus. Spatially resolved FA maps revealed that structural changes due to normal aging are not uniformly distributed^16,19^. However, spatially resolved evaluation of *FD* is possible, following the idea described in^48^. The challenge of extending our approach from a single value to a three dimensional spatially resolved FD map is less a question of feasibility than a question of computational efficiency.

## Conclusion

MRI based tractography of the brain is the most promising imaging technique for revealing cerebral white matter architecture. Changes in the structural complexity can reliably be measured with fractal analysis. Specifically the correlation dimension D_2_ is a reliable measurement to capture structural changes which was demonstrated for a large cohort of normal aging subjects. The sensitivity to structural changes due to aging is higher compared to changes in fractional anisotropy suggesting that fractal dimension might be a valuable biomarker for detecting structural changes in the brain. This is of exceptional interest for studying structural changes in healthy subjects as well as for pathologies provoking white matter changes.

## Methods

### Participants

All of the 85 participants (34 male between 21 and 74 years, 51 female between 18 and 81 years) had no history of chronic psychiatric or neurological diseases, brain or heart surgery. The Mini-Mental State Examination^49^ and the General Depression-scale (in German)^50^ ensured that all participants over 60 years were free from dementia and depression. Participant’s written informed consent was obtained according to the Declaration of Helsinki and the study was approved by the ethics committee of the Medical University of Graz.

### MRI data acquisition

Imaging data was acquired on a 3 T Siemens Skyra (Siemens Healtheneers, Erlangen, Germany) using a 32-channel head coil. Foam pads were used for the fixation of participants’ head during data acquisition. For diffusion weighted images, 50 transversal slices, oriented parallel to the AC-PC plane, were measured using a single-shot echo planar imaging sequence (TR = 6600 ms, TE = 95 ms, flip angle 90°, FoV = 240 mm, matrix size = 122 × 122 mm, 2 mm thickness, slice gap = 0.5 mm, GRAPPA acceleration factor = 2). One non-diffusion weighted image (b value = 0 s/mm²) and 64 diffusion sensitizing gradient directions were applied (b value = 1000 s/mm²). Additionally, structural images were obtained using a MPRAGE sequence (TR = 2530, TE = 2.07, TI = 900 ms, flip angle = 9°, Number of slices = 176, slice thickness = 1 mm, matrix = 256 × 256). Total scanning time was 13 minutes 33 seconds.

### MRI data processing

Diffusion data were analyzed using the FSL Software Library (v. 5.0.1) from the Oxford Centre for Functional MRI of the Brain (FMRIB), in a standard multi-step procedure including: (a) correction for eddy-currents and head-motion artefacts (b) removal of non-brain tissue based on the b = 0 images for every participant, using the Brain Extraction Tool (BET) (c) voxel-wise fitting of diffusion tensors and computation of fractional anisotropy (FA), using DTIfit. All of these steps are part of the FMRIB Diffusion Toolbox (FDT). For each subject, the T_1_-weighted image was registered to the b0-image using a rigid body transformation, implemented in SPM12 (vers. 6685). After binarization, the T_1_-image was segmented into gray matter, white matter and cerebrospinal fluid using SPM12 in native space (tissue types = 6, sampling distance = 3, segmented image voxel size = 1 × 1 × 1), resulting in individual white matter masks. These masks were applied to the FA-images in native space. The mean-FA value for the entire white matter was evaluated.

**Figure 6:**
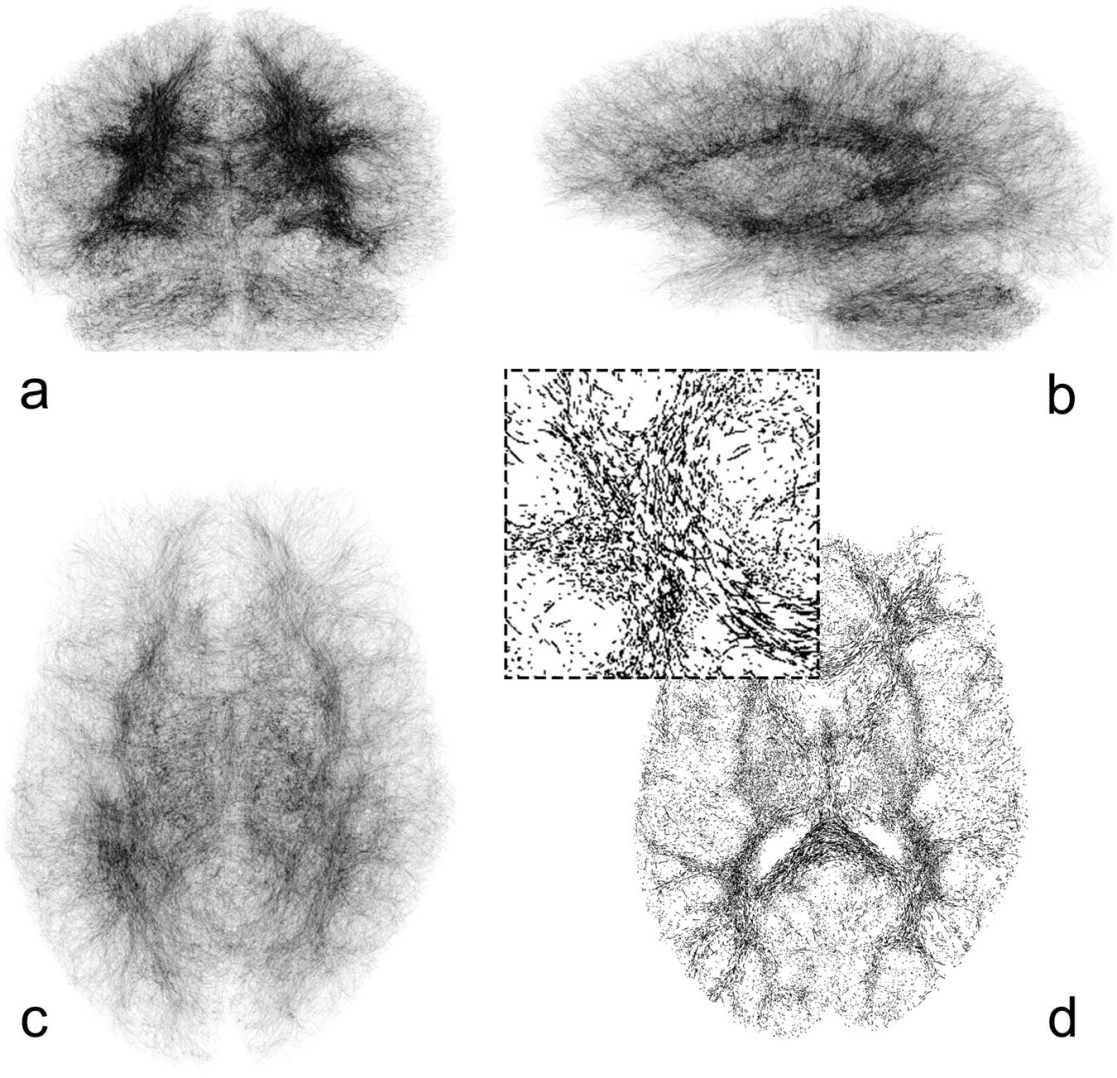
Tractography discretized on a 1024 × 1024 × 1024 grid. Rendered visualization in coronal (**a**), sagittal (**b**), and transversal (**c**) view. 10^4^ fibertracts were used for better visualization. Please note that 10^5^ fibertracts were used for *FD* calculation. Image d shows one central transversal slice with magnified view. Images are inverted for better visualization.

Tractography was performed using CSD, implemented in the MRTRIX 3.0 software package. The CSD method allows for estimating the fiber orientation distribution function (fODF) directly from the diffusion signal. We estimated the fODF based on an eighth-order harmonic function. A total number of 10^5^ tracks were kept constant for all data. The tracks, given though fulcrums in a three dimensional space, were discretized on a 1024 × 1024 × 1024 grid and displayed as binary image (Fig. 6). This approach is comparable to a binary version of super-resolution track-density mapping^42,43^.

### Fractal Analysis

The fractal dimension (*FD*) is often introduced by the Hausdorff dimension, a mathematical definition of fractals that cannot be directly solved. An approximation is possible, using the concept of self-similarity. An object with (*N)* segments scales with a length *(r)* thereby formulating a power law:

1 $$N={r}^{-FD}$$

hence,

2 $$FD=-\frac{\mathrm{log}\,N}{\mathrm{log}\,r},$$

where *FD* is the dimension of the scaling law. For non-fractal objects, *FD* equals the Euclidean dimension *(D = 1*, *2*, *3*, *… n)* that is one for a line, two for an area, and three for a volume. Fractal objects obey a metric scaling relation, where the exponent (the fractal dimension, *FD*) is not equal to the Euclidean dimension and is usually not an integer. In nature, a single exponent is often not enough to describe a complex structure and a spectrum of exponents is needed. The generalized dimensions or Rényi-dimensions *D*_*q*_ are defined according to:

3 $${D}_{q}=-\mathop{\mathrm{lim}}\limits_{\varepsilon \to 0}\frac{\mathrm{log}\,{\sum }_{i}\mu {({B}_{i})}^{q}}{(1-q)\mathrm{log}(\varepsilon )}=-\frac{{I}_{q}(\varepsilon )}{\mathrm{log}(\varepsilon )}$$

where *µ* is the probability density of elements in the i^th^ box *B*_*i*_, with a side length of ε. For q = 0, D_0_ is usually referred to as the capacity dimension that equals the box-counting dimension in that *µ* is the probability that the i^th^ Box *B*_*i*_ is populated. For q = 1, D_1_ is called the information dimension where the number of elements is counted for every Box *B*_*i*_. For q = 2, D_2_ is called the correlation dimension. These dimensions are theoretically related by the inequality

4 $${D}_{q}\ge {D}_{q+1}\,for\,q=[-\infty ,\infty ]$$

**Figure 7:**
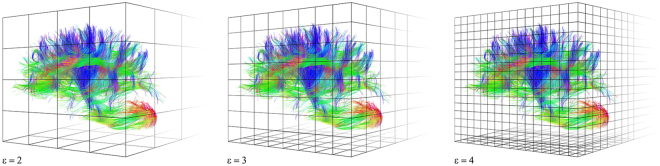
Principle of evaluating the fractal dimension using the box counting method. For estimating the fractal dimension, a structure to be analyzed is covered with boxes *B*_*i*_, with a side length of ε. The generalized dimensions are obtained for different q according to Eq. [3].

For an exact monofractal, the evaluated dimensions with varying q should be equal. In contrast, multifractals show a monotonic decreasing behavior in their spectrum of dimensions ${D}_{q}$ according to Eq. [4]. All calculations of the fractal dimension were done in a three dimensional space by covering the high-resolution tractograms with cubes. The multifractal spectrum was carried out within the range q = [0, 10] using cube side lengths ε in the range of n = [2, 8] given ε = 2^n^ (Fig. 7) This range, that was determined by simulations (data not presented here), has shown the best linear fit in the double logarithmic plot $1/(1-q)\mathrm{log}\,\sum _{i}\mu {({B}_{i})}^{q}$ versus $\,\mathrm{log}(\varepsilon )$ for all q. RMSE was evaluated for every q to reveal q showing the highest linearity. The highest linearity in the double logarithmic plot means that the data are closest to the power law (see Eq. 1) and hence, providing the best fractal description of the structure. Multifractal Analysis was implemented with MATLAB software (V 2015b, The MathWorks, Inc., MA, USA).

### Statistical Analysis

Linear models and polynomial models of 2^nd^ order were used to test for correlation between *FD* (D_2_) and FA with age. This allows a direct comparison of age-dependent changes of both, *FD* and FA. The Akaike Information Criterion was applied to select the most probable model. Evaluated Akaike Weights within the range [0, 1] provide the probability of the model. Gender differences were statistically tested with regards to the parameters age, *FD* (D_2_), FA, white matter brain volume, mean fiber length using an unpaired, two-tailed t-test. All statistical analyses were carried out using Origin Pro 9.0 (Northhampton, MA, USA)

### Data availability

All data are available from the corresponding author upon reasonable request.

### Ethical statement

All methods and experiments were performed in accordance with relevant national and international guidelines and regulations and were approved by the local ethics committee. All subjects gave written informed consent to participate in this study.
